# An International Survey of Health Care Providers Involved in the Management of Cancer Patients Exposed to Cardiotoxic Therapy

**DOI:** 10.1155/2015/391848

**Published:** 2015-08-02

**Authors:** Jeffrey Sulpher, Shrey Mathur, Daniel Lenihan, Christopher Johnson, Michele Turek, Angeline Law, Ellamae Stadnick, Franco Dattilo, Nadine Graham, Susan F Dent

**Affiliations:** ^1^The Ottawa Hospital Cancer Centre, University of Ottawa, Ottawa, ON, Canada K1H 8L6; ^2^Vanderbilt Heart and Vascular Institute, Nashville, TN 37232, USA; ^3^The Ottawa Hospital Division of Cardiology, University of Ottawa, Ottawa, ON, Canada K1H 8L6

## Abstract

Cardiotoxicity is the second leading cause of morbidity and mortality in cancer survivors. The objective of this international cardiac oncology survey was to gain a better understanding of current knowledge and practice patterns among HCPs involved in the management of cancer patients exposed to potentially cardiotoxic drugs. Between 2012 and 2013, we conducted an email-based survey of HCPs involved in the management of cardiac disease in cancer patients. 393 survey responses were received, of which 77 were from Canadian respondents. The majority of respondents were cardiologists (47%), followed closely by medical oncologists. The majority of respondents agreed that cardiac issues are important to cancer patients (97%). However, only 36% of total respondents agreed with an accepted definition of cardiotoxicity. While 78% of respondents felt that cardiac medications are protective during active cancer treatment, only 51% would consider prescribing these medications up-front in cancer patients. Although results confirm a high level of concern for cardiac safety, there continues to be a lack of consensus on the definition of cardiotoxicity and a discrepancy in clinical practice between cardiologists and oncologists. These differences in opinion require resolution through more effective research collaboration and formulation of evidence-based guidelines.

## 1. Introduction

Patients diagnosed with cancer today have improved five-year relative survival compared to just over a decade ago [1]. Treatment advances, including the introduction of targeted agents, continue to improve cancer survival. However, it is increasingly evident that targeted agents used in cancer therapy may negatively impact cardiovascular health [2].

Currently, cardiotoxicity is the second leading cause of morbidity and mortality in cancer survivors [3]. This has led to increasing interest by health care providers (HCPs) in developing multidisciplinary approaches to manage these patients. However, many issues in cardiac oncology remain unresolved, including a formally accepted definition of cardiac toxicity. There are few guidelines to assist in the management of patients with or at risk of cardiac toxicity. As a result, there are major knowledge gaps with limited consensus on the approach for diagnosis, management, and monitoring of cardiotoxicity. The objective of this international cardiac oncology survey was to gain a better understanding of current knowledge and practice patterns among HCPs involved in the management of cancer patients exposed to potentially cardiotoxic drugs. Additionally, we sought to obtain a census of clinical opinions concerning emerging cardiac oncology issues. Ultimately, this information will be used to inform clinical guidelines and to better standardize the diagnosis, management, and monitoring of cardiac toxicity related to cancer therapy.

## 2. Methods

Between 2012 and 2013, we conducted an email-based survey of HCPs involved in the management of cardiac disease in cancer patients. HCPs were identified using email directories from the Canadian Association of Medical Oncologists (CAMO), the Canadian Cardiovascular Society (CCS), the Canadian Cardiac Oncology Network (CCON), and the International Cardioncology Society (ICOS). The survey consisted of 14 base questions for international participants (ICOS) and an additional 30 questions for Canadian participants (CCON) related to cancer treatment-induced cardiotoxicity. The ICOS and CCON questionnaires were initially prepared and administered separately; the results were subsequently combined and analyzed together for this study. Questions contained multiple-choice options; some follow-up questions also allowed further elaboration. In addition to a series of short-stem questions, the CCON survey also contained two questions pertaining to a clinical case study. The case study described a 50-year-old female receiving trastuzumab for HER2 positive metastatic breast cancer. Her left ventricular ejection fraction (LVEF) at baseline was 55% but on repeat echocardiogram decreased to 30% with no cardiac symptoms. Respondents were asked to recommend further clinical management. A follow-up scenario was also included, where trastuzumab therapy was discontinued, and an angiotensin converting enzyme (ACE) inhibitor was initiated. Serial echocardiograms revealed an unchanged LVEF at 30%. The patient had no cardiac symptoms; however she was developing progressive metastatic disease. Respondents were again asked to recommend appropriate management.

The survey was developed and administered via the FLUIDS online system. A modified Dillman Total Design Survey Method was used to ensure maximal responses [4]. Descriptive data was collected and summarized.

## 3. Results

A total of 393 survey responses were received, of which 77 were from Canadian respondents. The majority of ICOS survey respondents were from the USA; there were also several respondents from Australia, Denmark, and Switzerland. The overall response rate was 25%. The majority of respondents were cardiologists (185/393, 47%), followed closely by medical oncologists (158/393, 40%) (Table 1). Overall, 55% of respondents were in academic practice (212/383). When considering the Canadian (CCON) respondents alone, the majority (66/77, 89%) were in academic practice. Thirty-five percent of respondents (26/77) had been practising for less than five years. Fifty-two percent (40/77) indicated that they had a dedicated cardiac oncology centre at their institution.

**Figure 1:**
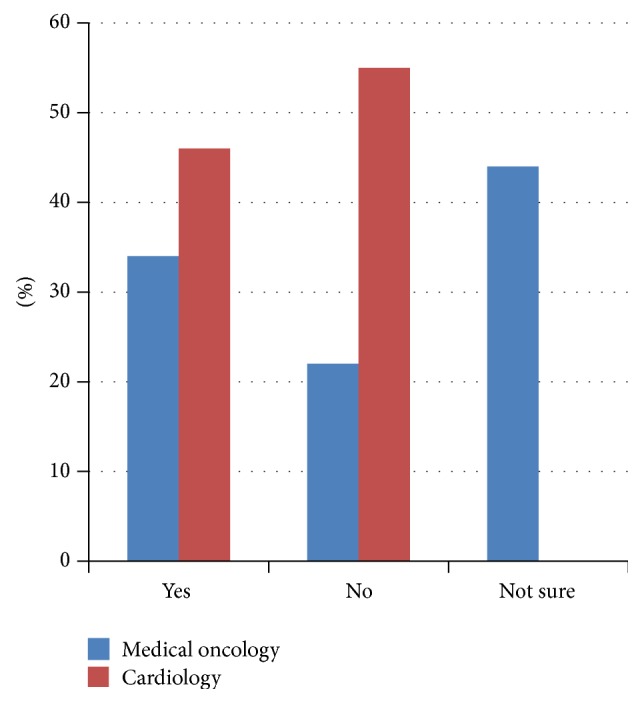
Is there a definition for “cardiac toxicity”? CCON results (*n* = 77).

**Figure 2:**
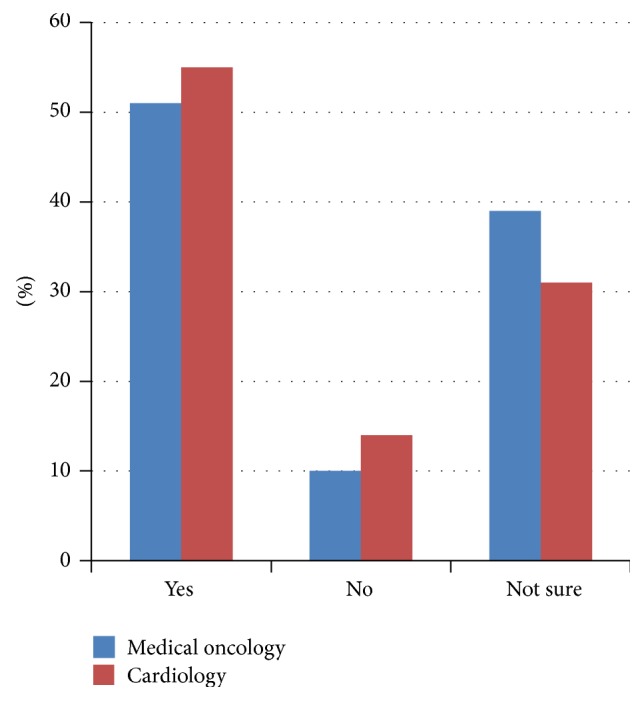
Are cardiac medications protective during active treatment? CCON results (*n* = 77).

**Figure 3:**
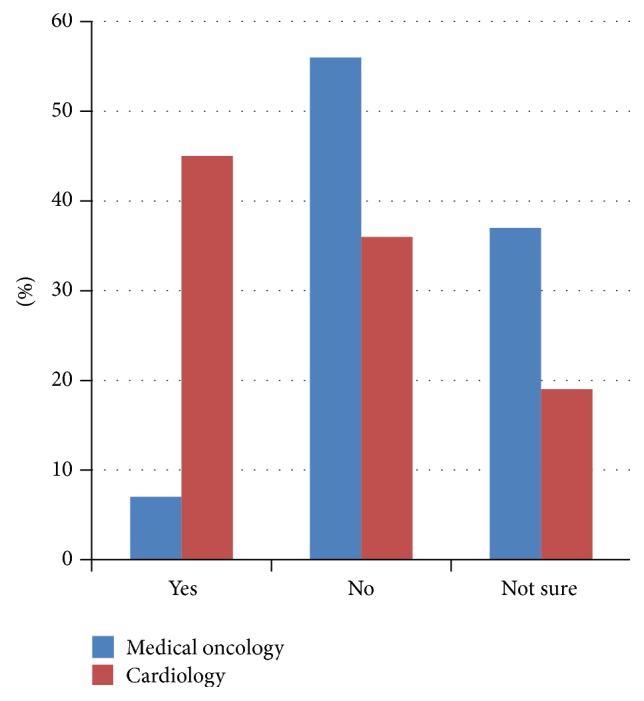
Would you prescribe cardiac medications to protect the heart during active cancer therapy? CCON results (*n* = 77).

The majority of respondents agreed that cardiac issues are important to cancer patients (381/393, 97%). Ninety-four percent felt that the diagnosis of cardiac disease had an impact on cancer prognosis (349/383) and 77% agreed that chemotherapy or radiation is an important risk factor for cardiac disease (301/393). However, only 36% of total respondents agreed with an accepted definition of cardiotoxicity (109/383). The majority of Canadian cardiologists felt that there is no formal definition of cardiotoxicity, while the majority of Canadian oncologists felt that there was an established definition (Figure 1). In spite of the high percentage (78%) of respondents who felt that cardiac medications are protective during active treatment (307/393), only 51% would consider prescribing these medications up-front in cancer patients (199/393). A large percentage of Canadian respondents answered “not sure” (29/77, 38%) to the protective effect of cardiac medications (Figure 2) and “not sure” (25/77, 32%) as to whether they would use them in clinical practice (Figure 3).

Referring to the clinical case study of the patient with decreased LVEF, the HCPs were asked “What would be your management of her trastuzumab therapy at this time?” Twenty percent of cardiologists chose the response “discontinue trastuzumab permanently,” while only 7% of oncologists chose this response. However, the response “discontinue trastuzumab, resume if EF normalizes” was chosen by 74% of oncologists, but by only 48% of cardiologists. In the follow-up question of unchanged LVEF in the presence of cancer progression, HCPs were asked “What management would you now recommend?” The results were scattered between the seven available options. The option “optimize ACE inhibitor, add beta blocker” was chosen by 52% of cardiologists and 22% of oncologists. The option “resume trastuzumab at reduced dose with serial EF” was chosen by 24% of cardiologists and 4% of oncologists. The option “other” was selected by 20% of cardiologists and 41% of oncologists.

## 4. Discussion

This international cardiac oncology survey was conducted to gain a better understanding of the knowledge base and clinical opinions of HCPs involved in the treatment of cancer patients being treated with potentially cardiotoxic therapy. To our knowledge, this is the first study of this kind in the field of cardiac oncology and highlights many controversial clinical issues within the field. The results affirm that opinions differ between cardiologists and oncologists regarding a formal definition of cardiotoxicity, as well as the diagnosis, management, and monitoring of oncology patients at risk of cardiovascular complications. At this time, there is no clear agreement in the literature on the definition of cancer therapy-related cardiotoxicity, and several historical definitions are in common use [5]. Recent consensus guidelines have recently been proposed in an attempt to clarify definitions; however it will take time to incorporate these recommendations into clinical practice [6]. Our results underscore the need for further collaboration between cardiologists and oncologists. Additionally, this survey demonstrated that there is a clear knowledge gap between cardiologists and oncologists in the appropriate clinical management of cancer patients who develop cardiotoxicity secondary to their cancer treatment. In the presented case study, more oncologists chose the evidence-based option [7] to “discontinue trastuzumab, resume if EF normalizes.” More concerning is that almost half (48%) of the cardiologists would not suggest resuming trastuzumab in these patients even with the normalization of their LVEF, thus depriving these patients of potentially lifesaving therapy.

The clinical opinions of the majority of respondents in this survey are supported by the available literature. The small percentage of respondents who felt that there is an established definition of cardiotoxicity (36%) is in agreement with work published by Albini and colleagues [8]. The finding that the majority of respondents agreed that chemotherapy or radiation is an important risk factor for cardiac disease is consistent with the conclusion by Suter and Ewer [9] that cancer treatments may induce cardiac dysfunction (7–34%), heart failure (1–4%), and arterial hypertension (up to 23%). Nearly four-fifths of respondents felt that cardiac medications may be protective during active treatment. Previous work by Yeh and colleagues reported that cardiac medications, such as ACE inhibitors and beta blockers, may be effective in patients being treated for cancer [10].

This study has several limitations. First, we were unable to compare results with other studies, as this survey was the first of its kind in cardiac oncology. Despite use of the modified Dillman Total Design Survey Method, only one-quarter of survey recipients responded. Response rates may be improved with use of personalized correspondence and monetary or unconditional incentives such as gift certificates [11]. The underlying reasons for survey nonresponse remain unclear and may contribute to nonresponse bias [12]. It is possible that nonresponding HCPs may not consider cardiac issues to be important in cancer treatment. Respondents were likely a highly selected sample of HCPs, since over half (52%, 40/77) indicated that they had access to a dedicated cardiac oncology clinic at their institution. Multiple iterations of this survey should be conducted to further validate the findings.

Second, the survey design forced respondents to select answers in a multiple-choice format, and respondents were limited to the choices provided. Furthermore, the order of the questions might affect the responses given. Some of the questions allowed for elaboration with free text, but these were not included in the analysis because the responses were so variable. Additionally, the ICOS and CCON groups were provided with separate surveys. Retrospectively combining these surveys proved difficult and limited the uniformity of results. For future investigations, all participants should be given a uniform survey over the same time period.

Cardiac oncology is a rapidly emerging but relatively new area of clinical medicine. It is encouraging to find a high level of concern for cardiac safety among health care providers treating cancer patients. Strikingly, there continues to be a lack of consensus on the definition of cardiotoxicity and a discrepancy in clinical practice between cardiologists and oncologists, the two specialties mostly involved in caring for cardiac oncology patients. These differences in opinion will need to be resolved through more effective research collaboration, formulation of evidence-based guidelines, and educational strategies to standardize the diagnosis, management, and monitoring of cardiac toxicity.

## Supplementary Material

Canadian Cardiac Oncology Network (CCON) survey (44 questions).

## Figures and Tables

**Table 1:** CCON and ICOS demographics.

Demographic	*N* = 393	%
Medical specialty		
Cardiology	185	47
Medical oncology	158	40
Other	50	13
Practice setting		
Academic	212	54
Community	114	29
Other	67	17

## Appendix

See Table 1.
