# Supplementary material for: An International Survey of Health Care Providers Involved in the Management of Cancer Patients Exposed to Cardiotoxic Therapy
Source: J Oncol. 2015 Aug 2;2015:391848. doi: 10.1155/2015/391848 (PMC4537762; doi:10.1155/2015/391848)
Supplement: Supplementary file 1 — Canadian Cardiac Oncology Network (CCON) survey (44 questions). [file 391848.f1.pdf]

What is your medical specialty?

- Cardiologist
- Medical Oncologist
- Radiation Oncologist
- Other, please specify... ________________________

In which province do you practice?

- Newfoundland Labrador
- Nova Scotia
- Prince Edward Island
- New Brunswick
- Quebec
- Ontario
- Manitoba
- Saskatchewan
- Alberta
- British Columbia
- Nunavut
- Northwest Territories
- Yukon

What is your practice setup?

- Academic institution
- Community centre
- Private practice
- Other, please specify... ________________________

How many years have you been in practice?

- 0-5 years
- 6-10 years
- 11-15 years
- 16-20 years
- > 20 years

Are you familiar with the terms 'cardiac oncology', 'cardio-oncology', 'cardioncology' or 'onco-cardiology'?

- Yes
- No
- Not sure

Do you have a dedicated cardiac oncology clinic at your institution?

- Yes
- No
- Not sure

If yes, have you referred a patient or seen a patient in such a clinic?

- Yes
- No

Do you feel that cardiac issues are clinically important in cancer patients?

- Yes

- No
- Not sure

In a patient with cancer, do you think diagnosing heart problems has any impact on their cancer prognosis?

- Yes
- No
- Not sure

How important is cardiology research in the cancer patient population?

- Not important
- Somewhat important
- Very important
- Essential
- Not sure

Is there an established definition of cardiotoxicity?

- Yes
- No
- Not sure

Is cardiotoxicity limited to cardiac muscle damage?

- Yes
- No
- Not sure

Do you think that chemotherapy or radiation treatment for cancer is an important risk factor for heart disease?

- Not important
- Somewhat important
- Very important
- Essential
- Not sure

In patients being actively treated for cancer, to what extent do you feel a cardiologist should be involved in their management?

- No need
- Rarely
- Sometimes
- Often
- Always needed
- Not sure

Do you think that cardiac medications protect the heart for patients being actively treated for cancer?

- Yes
- No
- Not sure

Do you think that cardioprotective medications should be considered in most patients being actively treated for cancer?

- Yes
- No
- Not sure

How important is it to consider possible cardiac problems during treatment for cancer?

- Not important
- Somewhat important
- Very important
- Essential
- Not sure

How important is it to consider possible cardiac problems in cancer survivors (patients with no active cancer who were treated at least 2-5 years ago)?

- Not important
- Somewhat important
- Very important
- Essential
- Not sure

Do you consider a recent history of cancer requiring systemic treatment to have an important impact on your choices of cardiac specific therapy?

- No impact
- Little impact
- Moderate impact
- Major impact
- Cardiac treatment should be minimized in patients being treated with cancer
- Not sure

Cardiac Monitoring

Do you follow a protocol for ejection fraction (EF) monitoring in cancer patients? If yes, please specify.

- Yes (please specify) ________________________
- No
- Not sure

What image modalities do you use to monitor EF in cancer patients?

- Echo (please specify EF %) ________________________
- MUGA (please specify EF %) ________________________
- Both (please specify EF % for both) ________________________
- Other, please specify... ________________________
- Not sure

Who do you refer to cardiology?

- All patients
- Only those with a known cardiac issue
- Patients with risk factors for cardiac disease
- Not sure
- Other, please specify... ________________________

Cancer Treatment

Do you interrupt cancer therapy for patients with low ejection fraction?

- Yes
- No
- Not sure

If yes, at which EF?

- ≤ 50%
- ≤ 45%
- ≤ 40%
- A drop ≥ 15%
- Other, please specify... ________________________

For what cardiac related clinical reason(s) would you interrupt cancer therapy? Please check all that apply.

- Decreased EF

- [ ] Uncontrolled hypertension
- [ ] Angina
- [ ] Other, please specify... ________________________
- [ ] Not sure

When do you resume cancer therapy?

- ( ) EF normal
- ( ) EF mildly reduced
- ( ) EF moderately reduced
- ( ) Clinical status
- ( ) Not sure

Cardiac Treatments

At what EF would you initiate cardiac treatment?

- ( ) All abnormal EF values
- ( ) If EF does not rise after cancer therapy discontinuation
- ( ) If repeat EF remains abnormal
- ( ) Not sure
- ( ) Other, please specify... ________________________

What treatment(s) do you initiate first?

- ( ) Angiotensin converting enzyme inibitors (ACEi) first
- ( ) Beta Blockers (BB) first
- ( ) Not sure
- ( ) Other, please specify... ________________________

Do you routinely prescribe aldosterone antagonists for cancer-related cardiotoxicity?

- ( ) Yes
- ( ) No
- ( ) Not sure

Do you investigate for coronary artery disease? If yes, specify with which modality.

- ( ) Yes (please specify modality) ________________________
- ( ) No
- ( ) Not sure

Long Term Follow Up

Do you follow all cancer patients with a history of cardiac toxicity?

- ( ) Yes
- ( ) No

If yes, how long do you follow these patients for?

- ( ) 0-3 months
- ( ) 3-6 months
- ( ) 6-12 months
- ( ) > 12 months
- ( ) Other, please specify... ________________________

Do you continue cardiac medications in all patients with a history of cardiac toxicity?

- ( ) Yes
- ( ) No
- ( ) Depends on patient's cardiac risk factors
- ( ) Not sure

If yes, for how long?

- 0-3 months
- 3-6 months
- 6-12 months
- > 12 months
- Indefinitely

How often do you monitor EF in patients with early stage breast cancer?

- Every 3 months
- Every 6 months
- Every 9 months
- Every 12 months
- Other, please specify... ________________________

How often do you monitor EF in patients with metastatic breast cancer?

- Every 3 months
- Every 6 months
- Every 9 months
- Every 12 months
- Other, please specify... ________________________

Cases

A 62 year old female is diagnosed with metastatic breast cancer to bone. Her tumor is estrogen/progesterone receptor negative and Her-2/neu receptor positive. She is initiated on systemic chemotherapy (docetaxel) and trastuzumab administered every 3 weeks. Her initial transthoracic echocardiogram reveals an ejection fraction of 53%. How often would you monitor this patient's ejection fraction while on trastuzumab therapy?

- Every 3 months
- Every 6 months
- Every 9 months
- Every 12 months
- Not sure
- Other, please specify... ________________________

What is the optimal method of monitoring this patient's ejection fraction?

- Echocardiogram
- MUGA
- Not sure
- Other, please specify... ________________________

A 50 year old female has received 12 cycles of trastuzumab therapy for Her-2/neu positive metastatic breast cancer to liver. Her ejection fraction at baseline was 55%, but on repeat echocardiogram decreased to 30%. She has no cardiac symptoms. What would be your management of her trastuzumab therapy at this time?

- Continue trastuzumab therapy at full dose
- Continue trastuzumab therapy at reduced dose
- Discontinue trastuzumab therapy and monitor EF. Resume trastuzumab if EF normalizes
- Discontinue trastuzumab therapy permanently
- Not sure
- Other, please specify... ________________________

Trastuzumab therapy is discontinued, and an ACE inhibitor is initiated. Serial echocardiograms reveal an unchanged EF at 30%. The patient has no cardiac symptoms, however she is developing progressive metastatic disease. What management would you now recommend?

- Resume trastuzumab at full dose with serial echocardiograms
- Resume trastuzumab at reduced dose with serial echocardiograms

- Continue to hold trastuzumab therapy with serial echocardiograms
- Pursue investigations for coronary artery disease
- Optimize ACE inhibitor and add a beta blocker
- Not sure
- Other, please specify... ________________________

A 58 year old male is receiving adjuvant infusional 5-fluorouracil for resected stage III colorectal carcinoma. He develops sudden chest pain and nausea, and presents to the emergency department. A 12 lead electrocardiogram reveals inferior ST segment elevation. He is managed medically with complete resolution of symptoms. A subsequent angiogram reveals no evidence of coronary artery disease. A follow-up echocardiogram reveals an EF of 58%. What would you now recommend for adjuvant chemotherapy?

- Resume 5-fluorouracil at full dose
- Resume 5-fluorouracil at full dose but administer with cardiac monitoring
- Resume 5-fluorouracil at a reduced dose
- Change chemotherapy to oral capecitabine
- Change chemotherapy to intravenous raltitrexed
- Discontinue adjuvant chemotherapy
- Not sure
- Other, please specify... ________________________
